# The relationship of peripheral blood lncRNA-PVT1 and miR-146a levels with Th17/Treg cytokines in patients with Hashimoto’s thyroiditis and their clinical significance

**DOI:** 10.17305/bb.2024.10237

**Published:** 2024-10-01

**Authors:** Yi-nan Li, Jingxue Shen, Yinglan Feng, Yingyan Zhang, Yusi Wang, Xinyu Ren

**Affiliations:** 1 Department of Endocrinology 2, Central Hospital Affiliated to Shenyang Medical College, Tiexi District, Shenyang, Liaoning Province, China; 2 Department of Internal Medicine 1, Tacheng People’s Hospital of Xinjiang, Tacheng, Xinjiang, China

**Keywords:** Long noncoding RNA, plasmacytoma variant translocation 1 (PVT1), miR-146a, Hashimoto’s thyroiditis (HT), T helper 17 (Th17), Treg, correlation

## Abstract

Hashimoto’s thyroiditis (HT) is a prevalent autoimmune disease. We investigated the relationship of peripheral blood long noncoding RNA-plasmacytoma variant translocation 1 (lncRNA-PVT1) and microRNA (miR)-146a levels with Th17/Treg-related cytokines in HT patients and their clinical significance. Correlations of lncRNA-PVT1 and miR-146a with Th17/Treg-related cytokines were analyzed, and its clinical value in diagnosing HT was assessed. Results showed reduced lncRNA-PVT1 and IL-10 levels and increased miR-146a and IL-17 levels in HT patients. LncRNA-PVT1 negatively interrelated with miR-146a, IL-17, IL-23, and IL-6, and positively interrelated with IL-10; miR-146a positively correlated with IL-17, IL-23, and IL-6, but negatively correlated with IL-10 in HT patients. The area under the curve (AUC) of lncRNA-PVT1 and miR-146a levels for diagnosing HT were 0.822 and 0.844, respectively (sensitivity 88.73% and 86.62%, specificity 67.02% and 69.15%, cut-off values 0.76 and 2.73), with their combined detections yielding a higher AUC. Patients with poorly expressed lncRNA-PVT1 and highly expressed miR-146a had elevated HT incidence. LncRNA-PVT1 and miR-146a levels were also found to be an independent influencing factor for HT occurrence. Our findings suggest that HT patients have low peripheral blood lncRNA-PVT1 expression and high miR-146a expression. LncRNA-PVT1 and miR-146a level changes were correlated with Th17/Treg cytokine imbalance and could be a potential diagnostic tool and independent influencing factor for HT.

## Introduction

Hashimoto’s thyroiditis (HT) is widely recognized as the most prevalent autoimmune disorder and the primary etiology of hypothyroidism, which is characterized by the infiltration of lymphocytes, resulting in damage to the thyroid gland [1]. Importantly, elevated levels of thyroid peroxidase antibody (TPOAb) and thyroglobulin antibody (TGAb) above 60 IU/mL are contributing factors to this condition [2]. In addition, among the various subpopulations of CD4+ T-cells, T helper 17 (Th17) and regulatory T cell (Treg) play crucial roles in maintaining immune homeostasis in the body, despite their contrasting functions [3]. Notably, Th17/Treg imbalance is directly bound up with TPOAb and TgAb [4]. The impaired capacity of Treg to suppress the proliferative ability of T cells against the self can disrupt immune balance [5]. Despite the considerable amount of research conducted, the underlying mechanisms responsible for HT remain incompletely elucidated, and additional investigations are warranted to enhance our understanding and uncover novel therapeutic targets for the prevention and management of HT [6].

Long noncoding RNAs (lncRNAs) refer to a specific class of RNA molecules greater than 200 base pairs in length that are cell- and tissue-specific [7]. LncRNAs play a prominent role in immune cell differentiation, proliferation, and apoptosis through the modulation of target gene expression, and are essential for regulating immune responses and maintaining immune balance [8–10]. The lncRNA-plasmacytoma variant translocation 1 (PVT1) is situated at 8q24. 21, and exhibits a remarkable correlation with the onset and progression of cancer, as well as displaying aberrant expression patterns in autoimmune disorders [11]. It has been documented that lncRNA-PVT1 pitches in the proliferation and differentiation of Th17 cells by manipulating the NOTCH pathway [12]. LncRNA-PVT1 overexpression upregulates TNF receptor-related factor TRAF6 expression by directly targeting microRNA (miR)*-*146a, and the interaction between lncRNA-PVT1 and miR-146a has been found to impact the Treg autophagy, suggesting that lncRNA-PVT1 and miR-146a are implicated in the modulation of the Th17/Treg-related cytokine imbalance and that they are crucial for maintaining immune homeostasis [13]. Currently, there are limited reports available regarding the specific target molecules and the potential pathways tied up with lncRNA-PVT1 and miR-146a in the onset and progression of HT. Based on this context, this study aimed to probe the relationships of peripheral blood lncRNA-PVT1 and miR-146a expression levels with Th17/Treg-related cytokines in patients with HT and their clinical significance.

## Materials and methods

### Patient information

A total of 200 patients with HT who were admitted to Central Hospital Affiliated to Shenyang Medical College between January 2021 and April 2023 were retrospectively consecutively selected. In light of the inclusion and exclusion criteria, 142 HT patients, all of whom exhibited hypothyroidism or subclinical hypothyroidism and without clinical treatment, were finally included in the HT group. Simultaneously, 94 healthy volunteers who underwent thyroid function examination in Central Hospital Affiliated to Shenyang Medical College and exhibited normal results were selected as the normal control (NC) group. The clinical data of all subjects were complete.

### Inclusion and exclusion criteria

The following inclusion criteria for the HT group were defined based on the diagnostic criteria for HT outlined in the Chinese Guidelines for Diagnosis and Treatment of Thyroid Diseases (Chinese Society of Endocrinology, Chinese Medical Association, 2008 version): patients with 1) persistent positivity of thyroid autoantibodies (such as TPOAb and TGAb) tested by serological testing; 2) diffuse goiter, especially accompanied by enlargement of isthmic vertebral lobe with a tough texture; 3) typical alteration of thyroid hypoechoic lesions tested by thyroid ultrasound; and 4) thyroid stimulating hormone (TSH) higher than normal range (0.27–4.2 mIU/L) tested by thyroid function testing. The NC group met the following criteria: 1) all indicators of blood routine in peripheral blood specimens were within the normal range; 2) matched with the HT group in terms of sex and age; 3) serological testing for free triiodothyronine (FT3), free thyroxine (FT4), TSH, TPOAb, and TGAb and other thyroid functions and autoantibodies, were within the normal range; and 4) no history of thyroid-related diseases or autoimmune diseases.

Exclusion criteria included: (1) heart, liver, kidney, and other important organ dysfunctions; (2) thyroid dysfunction caused by simple goiter or other causes; (3) fever, pregnancy, and lactating women; (4) complicated with malignant tumors, other autoimmune, endocrine diseases, and systemic diseases (such as rheumatoid arthritis, systemic lupus erythematosus, inflammatory diseases, asthma, or other allergic diseases); and (5) history of infection or immunosuppressive drug administration recently.

### Data collection

General information of all the subjects, such as age, sex, height, weight, smoking history, drinking history, blood pressure, blood lipids, comorbidities, and thyroid function test results (TSH, FT3, FT4, TPOAb, and TGAb) were collected. Fasting elbow venous blood was obtained and sent to each laboratory as required.

### Thyroid function test

The fasting venous blood (3 mL) was collected from subjects to acquire serum samples. A chemiluminescence immunoassay analyzer (ADVIA Centaur CP chemiluminescence immunoassay analyzer, Huasing Bio, Beijing, China) was utilized for the detection of TSH, FT3, FT4, TPOAb, and TGAb levels.

### Enzyme-linked immunosorbent assay (ELISA) assay

The extracted 2 mL of fasting elbow venous blood was subjected to agglutination at room temperature for 30 min and then centrifugation at 3200 r/min for 8 min. The serum was separated and placed in a refrigerator at –80 ^∘^C. ELISA kits (Gersion, Beijing, China) were utilized to assess interleukin (IL)-17 (QS40180), IL-23 (QS40196), IL-6 (QS40049), and IL-10 (QS450711) levels of serum samples on a Multiskan Mk3 automatic microplate reader (Thermo Fisher Scientific, Waltham, MA, USA).

### RNA isolation and reverse transcription-quantitative polymerase chain reaction (RT-qPCR) assay

Serum samples were preserved in 1.5 mL RNase-free microcentrifuge tubes for RNA concentration determination within one week. Total RNA was extracted from serum using TRIzol reagent (Thermo Fisher Scientific). LncRNA-PVT1 and miR-146a were separately reverse transcribed utilizing a reverse transcription kit (Promega, Madison, WI, USA) and the stem-loop method (QIAGEN, Shanghai, China). The number of complementary DNA was determined using a Nano-Drop 2000 spectrophotometer (Thermo Fisher Scientific), and its quality was determined by 2% agarose gel electrophoresis and ethyl bromide staining. Glyceraldehyde-3-phosphate dehydrogenase and U6 served as internal references for RT-qPCR. The primer sequences are listed in Table 1. The reaction conditions were as follows: denaturation at 95^∘^C for 10 min, 40 cycles of 95^∘^C for 15 s, 60^∘^C for 15 s, and 75^∘^C–95^∘^C extension period for 1^∘^C/20 s. The relative expression levels of serum lncRNA-PVT1 and miR-146a were quantified using the 2^−ΔΔCt^ method.

**Table 1:** Primer sequences

**Gene**	**Forward (5′-3′)**	**Reverse (5′-3′)**
lncRNA-PVT1	GCCCCTTCTATGGGAATCACTA	GGGGCAGAGATGAAATCGTAAT
miR-146a	CAGCTGCATTGGATTTACCA	GCCTGAGACTCTGCCTTCTG
*GAPDH*	TGCACCACCAACTGCTTAG	GGATGCAGGGATGATGTTC
*U6*	CTCGCTTCGGCAGCACA	AACGCTTCACGAATTTG

### Ethical statement

This study was reviewed and approved by the Ethics Committee of Central Hospital Affiliated to Shenyang Medical College and complied with the Declaration of Helsinki. All participants and their families were fully informed of the purpose of the study and signed the informed consent prior to the study.

### Statistical analysis

Data were statistically analyzed and graphed using SPSS 21.0 (IBM, Armonk, NY, USA) and GraphPad Prism 6.0 (GraphPad, San Diego, CA, USA). The Kolmogorov–Smirnov test was conducted to test for normal distribution, and measurement data that conforming to normal distribution were expressed as mean ± standard deviation (SD), with *t*-test used for comparisons between groups; measurement data of non-normal distribution were presented as median (interquartile range), namely P50 (P25, P75), with the Mann–Whitney *U* test applied for comparisons between groups. Counting data were expressed as a number of cases and percentages, with the chi-square test implemented for comparisons between groups. Continuous data conforming to normal distribution were analyzed using the Pearson correlation coefficient. The receiver operating characteristic (ROC) curve was plotted to evaluate the diagnostic efficacy of the parameters and to obtain the cut-off values, with the area under curve (AUC) used to analyze the diagnostic value of lncRNA-PVT1 and miR-146a for HT. Logistic regression analysis was used to evaluate the influencing factors for HT occurrence. First, univariate analysis was performed on each risk factor. Then, risk factors with *P* < 0.05 were included in multivariate logistic regression analysis, and the Enter method was used to screen independent variables. *P* was a two-sided test, with *P* < 0.05 deemed to be statistically significant.

## Results

### Clinical baseline characteristics

No prominent differences were observed between the two groups in terms of age, sex, body mass index, smoking history, drinking history, and comorbidities (all *P* > 0.05). FT3 and FT4 levels were reduced (all *P* < 0.05), whereas TSH, TPOAb, TGAb, and the urinary iodine levels were elevated in the HT group vs the NC group (all *P* < 0.05, Table 2).

**Table 2:** Comparisons of clinical baseline characteristics

**Characteristics**	**NC group (*n* ═ 94)**	**HT group (*n* ═ 142)**	***P* value**
Sex (male/female)	11/83	16/126	0.918
Age (years)	33.62 ± 8.57	35.19 ± 7.67	0.143
BMI (kg/m^2^)	23.51 ± 2.39	24.04 ± 2.17	0.079
Positive smoking history (*n*, %)	29 (30.85)	41 (28.87)	0.745
Positive drinking history (*n*, %)	33 (35.11)	46 (32.39)	0.666
Diabetes mellitus (*n*, %)	18 (19.15)	28 (19.72)	0.914
Hypertension (*n*, %)	20 (21.28)	33 (23.24)	0.724
Dyslipidemia (*n*, %)	24 (25.53)	37 (26.06)	0.928
UI (µg/L)	161.37 ± 42.96	186.36 ± 37.49	<0.001
TSH (mlU/L)	1.77 ± 0.51	31.65 (10.18, 55.80)	<0.001
FT3 (pmol/L)	5.22 ± 1.23	4.62 ± 1.35	<0.001
FT4 (pmol/L)	16.87 ± 5.67	14.59 ± 4.06	<0.001
TPOAb (IU/mL)	19.92 (15.24, 57.57)	377.47 ± 159.48	<0.001
TGAb (IU/mL)	41.17 ± 14.52	191.70 ± 83.14	<0.001

Counting data are expressed as cases and percentages, and the chi-square test was applied for comparisons between groups. Measurement data of normal distribution are expressed as mean ± SD, with *t*-test adopted for comparisons between groups. Measurement data with non-normal distribution are expressed as median (IQR), followed by Mann–Whitney *U* test for intergroup comparisons. BMI: Body mass index; UI: Urinary iodine; TSH: Thyroidstimulating hormone; FT3: Free triiodothyronine; FT4: Free thyroxine; TPOAb: Thyroid peroxidase antibody; TGAb: Anti-thyroglobulin antibody; NC: Negative control group.

### Reduced peripheral blood lncRNA-PVT1 expression and elevated miR-146a expression were observed in HT patients

**Figure 1.**
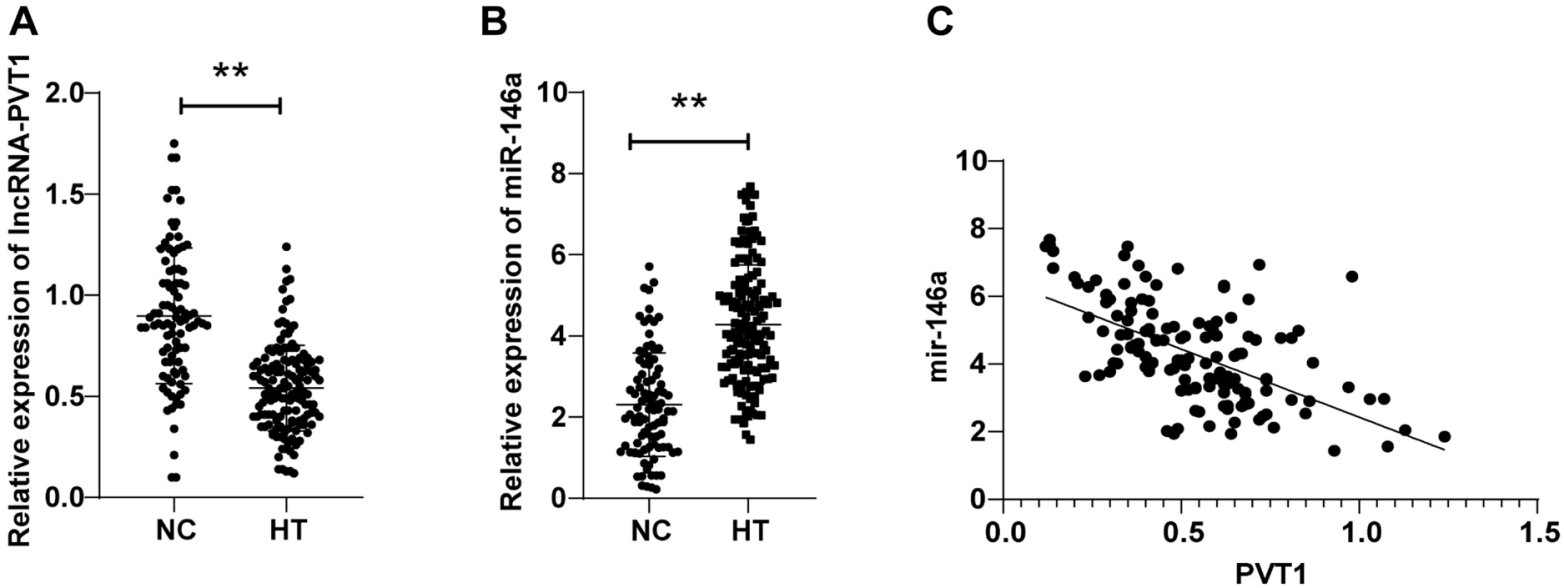
**Comparisons of peripheral blood lncRNA-PVT1 and miR-146a expression levels in HT patients.** (A and B) RT-qPCR to measure peripheral blood lncRNA-PVT1 and miR-146a expression patterns of the HT and NC groups; (C) Pearson correlation coefficient to analyze the correlations between peripheral blood lncRNA-PVT1 and miR-146a of HT patients. Data are expressed as mean ± SD. Comparisons between the two groups were performed by *t*-test. *P < 0.05, **P < 0.01. HT: Hashimoto’s thyroiditis; SD: Standard deviation; NC: Normal control group; RT-qPCR: Reverse transcription-quantitative polymerase chain reaction.

LncRNA-PVT1 expression levels were (0.90 ± 0.33) in the NC group and (0.54 ± 0.21) in the HT group, with lncRNA-PVT1 levels in the HT group dramatically decreased compared to the NC group (*P* < 0.01, Figure 1A). miR-146a expression levels were (2.30 ± 1.27) in the NC group and (4.28 ± 1.46) in the HT group, with miR-146a levels in the HT group remarkably hoisted relative to the NC group (*P* < 0.01, Figure 1B). Furthermore, as reflected by Pearson analysis, peripheral blood lncRNA-PVT1 was adversely correlated with miR-146a levels in HT patients (r ═ −0.585, *P* < 0.01, Figure 1C). The aforesaid results unveiled that peripheral blood lncRNA-PVT1 expression was reduced and miR-146a expression was boosted in HT patients, and they were significantly negatively correlated.

### Peripheral blood Th17/Treg-related cytokine imbalance in HT patients

**Figure 2.**
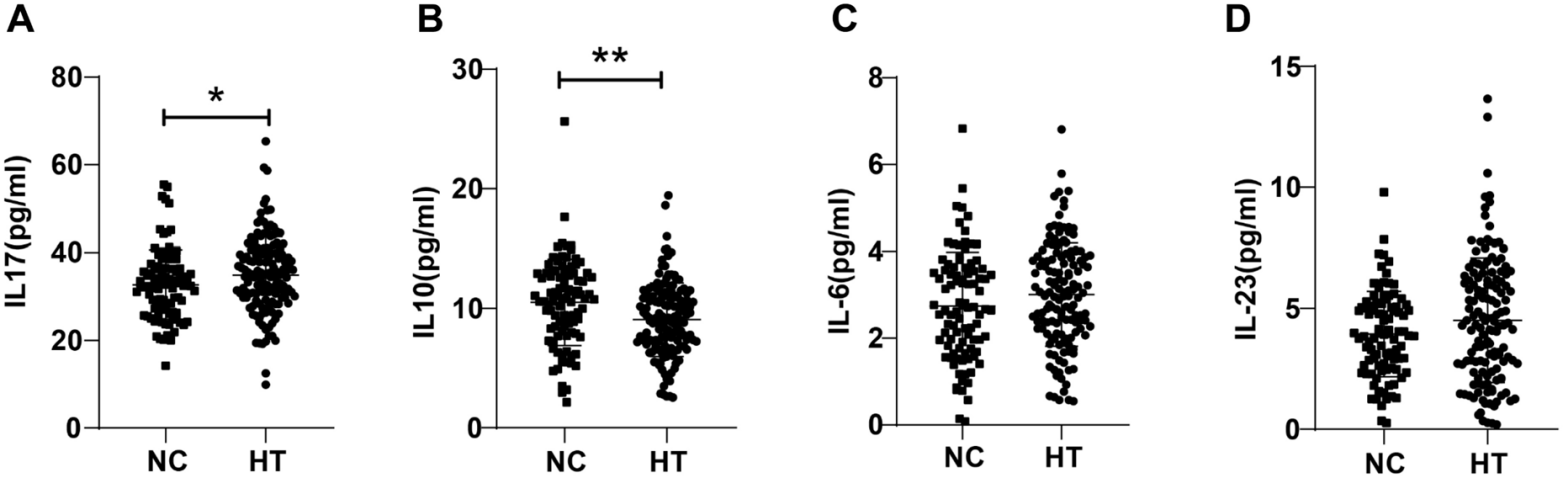
**Comparisons of peripheral blood Th17/Treg-related cytokine levels in HT patients.** (A–D) Th17/Treg-related cytokine (IL-17, IL-23, IL-6, IL-10) levels in the HT and NC groups were measured by ELISA. Comparisons of data between groups: *P < 0.05, **P < 0.01. HT: Hashimoto’s thyroiditis; NC: Normal control group; ELISA: Enzyme-linked immunosorbent assay.

As reflected by ELISA results, relative to the NC group, HT group exhibited elevated peripheral blood IL-17 (*P* < 0.05, Figure 2A) and abated IL-10 (*P* < 0.01, Figure 2B) levels. However, there were no distinct differences in IL-23 and IL-6 levels between the patients of the two groups (all *P* > 0.05, Figure 2C and 2D).

### Correlations of lncRNA-PVT1 and miR-146a levels with Th17/Treg-related cytokines

Pearson analysis elicited that peripheral blood lncRNA-PVT1 was inversely interrelated with IL-17, IL-23, and IL-6 levels, and favorably relevant to IL-10 level in HT patients (all *P* < 0.05). Peripheral blood miR-146a level of HT patients displayed positive correlations with IL-17, IL-23, and IL-6 levels, and adverse interrelations with IL-10 level (Table 3, all *P* < 0.05).

**Table 3:** Correlation analyses of lncRNA-PVT1 and miR-146a expression levels with Th17/Treg cytokines

**Indicator**	**lncRNA-PVT1**		**miR-146a**	
	***r* value**	***P* value**	***r* value**	***P* value**
IL-17	−0.598	<0.001	0.359	<0.001
IL-23	−0.266	0.001	0.218	0.009
IL-6	−0.254	0.002	0.178	0.035
IL-10	0.63	<0.001	−0.503	<0.001

### Peripheral blood lncRNA-PVT1 and miR-146a aided in HT diagnosis

**Figure 3.**
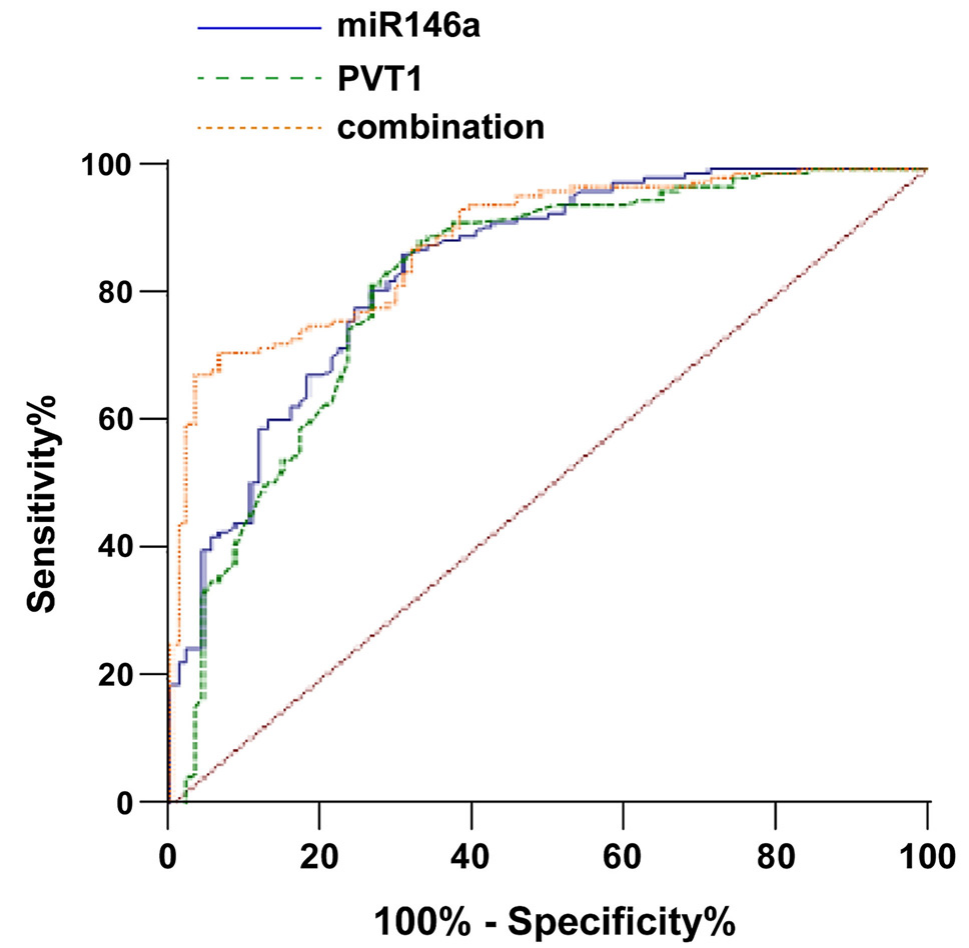
**ROC curve analysis of the value of lncRNA-PVT1, miR-146a alone and their combination for HT diagnosis.** ROC: Receiver operating characteristic; HT: Hashimoto’s thyroiditis.

The diagnostic value of lncRNA-PVT1 and miR-146a in HT was studied. ROC curve was plotted, with the results revealing that the AUC of lncRNA-PVT1 for HT diagnosis was 0.822 (cut-off value 0.76, sensitivity 88.73%, and specificity 67.02%). The AUC of miR-146a for HT diagnosis was 0.844 (cut-off value 2.73, sensitivity 86.62%, and specificity 69.15%) (Figure 3). In light of the cut-off value of lncRNA-PVT1 and miR-146a, the study subjects were assigned to high/low expression groups, with HT incidence compared between the two groups. The results manifested that HT incidence in the lncRNA-PVT1 low-expression group was 80.25%, which was higher than that in the lncRNA-PVT1 high-expression group (20.25%), and HT incidence in the miR-146a high-expression group was 80.92%, which was enhanced compared to the miR-146a low-expression group (22.62%) (all *P* < 0.05) (Table 4). Additionally, the AUC of the combination of the two demonstrated a conspicuous elevation compared to that of lncRNA-PVT1 and miR-146a alone (all *P* < 0.05, Table 5).

**Table 4:** Comparison of HT occurrence with different lncRNA-PVT1 and miR-146a expression levels

**Indicator**	**Expression**	**HT (cases)**	**NC (cases)**	**Total (cases)**	**HT incidence (%)**	***P* value**
lncRNA-PVT1	Low expression group	126	31	157	80.25	<0.001
	High expression group	16	63	79	20.25	
miR-146a	Low expression group	19	65	84	22.62	<0.001
	High expression group	123	29	152	80.92	

HT: Hashimoto’s thyroiditis; NC: Normal control group.

**Table 5:** Diagnostic value of lncRNA-PVT1 and miR-146a alone and their combination for HT

**Indicator**	**AUC**	**95% CI**	**Sensitivity**	**Specificity**
miR-146a	0.844	0.792∼0.888	86.62	69.15
lncRNA-PVT1	0.822	0.767∼0.869	88.73	67.02
Combination	0.888	0.840∼0.925	71.13	93.62
miR-146a∼lncRNA-PVT1	*P* ═ 0.550			
miR-146a*∼*combination	*P* ═ 0.016			
lncRNA-PVT1*∼*combination	*P* ═ 0.005			

AUC: Area under the curve; CI: Confidence interval; HT: Hashimoto’s thyroiditis.

### Peripheral blood lncRNA-PVT1 and miR-146a were independent influencing factors for HT occurrence

To accurately assess the effects of lncRNA-PVT1 and miR-146a on the occurrence and development of HT, we included lncRNA-PVT1 and miR-146a levels, and Th17/Treg-related cytokine levels (IL-17, IL-23, IL-6, and IL-10) into the univariate logistic regression analysis. LncRNA-PVT1, miR-146a, IL-17, and IL-10 levels were the influencing factors for HT occurrence. Subsequently, multivariate logistic regression analysis was performed with indicators with *P* < 0.05 in univariate regression analysis as independent variables. The results revealed that highly expressed miR-146a was an independent risk factor for HT occurrence, and high expression of lncRNA-PVT1 was a protective factor for HT occurrence (Table 6).

**Table 6:** Analyses of factors influencing HT occurrence

**Indicator**	**Univariate logistic regression analysis**			**Multivariate logistic regression analysis**		
	***P* value**	**OR **	**95% CI**	***P* value**	**OR **	**95% CI**
lncRNA-PVT1	<0.001	0.007	0.002∼0.029	<0.001	0.016	0.003∼0.077
miR-146a	<0.001	2.909	2.188∼3.867	<0.001	2.658	1.914∼3.691
IL-17	0.049	1.033	1.000∼1.067	0.475	0.984	0.943∼1.028
IL-23	0.066	1.118	0.993∼1.259	–	–	–
IL-6	0.099	1.203	0.966∼1.498	–	–	–
IL-10	0.002	0.874	0.803∼0.950	0.221	1.077	0.956∼1.213

CI: Confidence interval; OR: Odds ratio; HT: Hashimoto’s thyroiditis; IL: Interleukin.

## Discussion

HT is a widely prevalent autoimmune disease that is observed on a global scale, distinguished by the circulating autoantibodies against TPOAb and TGAb and the thyroid chronic inflammation [14]. HT commonly presents as hypothyroidism, which occurs due to the immune-mediated destruction of thyroid cells, and the pathogenesis of HT is a multifaceted process involving various factors such as environment, cytokines, genetic inclination, trace elements immune factors, as well as miRNA and DNA [15]. Among them, humoral and cellular immunity are crucial factors in HT development [16]. The prevalence of HT is on the rise, particularly among Caucasians, with an estimated rate of approximately 5%, but the underlying mechanisms responsible for HT development remain incompletely elucidated [17]. Therefore, it is essential to elucidate the pathogenesis of HT. Our study highlighted that peripheral blood lncRNA-PVT1 was poorly expressed, miR-146a was highly expressed in HT patients, and lncRNA-PVT1 and miR-146a were significantly correlated with Th17/Treg-related cytokine imbalance and could help HT diagnosis and were independent influencing factors for HT occurrence.

Th17 is characterized by the secretion of IL-17, and is known as the Th17 cell subset. Among them, IL-17 is the primary cytokine responsible for inducing inflammatory effects [18]. The main pathological manifestation of HT involves the destruction of thyroid follicular epithelial cells, and IL-17 indirectly participates in the inflammatory response by stimulating the inflammatory mediators and T-cell proliferation, leading to the inflammatory response of thyroid tissues, generation of thyroid autoantibodies, and tissue damage [4]. A relevant study has demonstrated the notable role of the pro-Th17 differentiation cytokine IL-23 in the maintenance, survival, and expansion of Th17 cells [19]. Treg cells are a subset of T cells that can specifically express the repressor gene FOXP3, and immunomodulatory effector cells that can secrete IL-10, transforming growth factor β and other anti-inflammatory factors. The secretion of IL-10 by Treg cells is considered a potent anti-inflammatory agent in numerous diseases, thereby holding substantial research implications for the therapeutic outlook of various chronic conditions [20–22]. Meanwhile, IL-10 can hinder lymphocyte infiltration in the thyroid gland and the destruction of thyroid follicular cells by inhibiting the secretions of pro-inflammatory factors and cytotoxic molecules (IL-1 and TNF-α) by IFN-γ-activated monocytes, with a certain protective effect, making IL-10 a promising candidate for an evaluation index associated with autoimmune diseases. IL-17 and IL-23 levels are rising in the thyroid tissue of HT patients [23]. IL-10 serves to manipulate the activity of Th17 and exerts a protective function in mitigating the damage to the thyroid gland [5]. Interestingly, our study results revealed that the peripheral blood IL-17 level in HT patients was boosted and IL-10 level was reduced.

LncRNAs have been identified as potential crucial regulators in the adaptive and innate immune response systems [24]. Among them, lncRNA-PVT1 significantly impacts the functionality of T cells [25]. Yu et al. [12] have unveiled that lncRNA-PVT1 is diminished in thrombocytopenia, while the upregulation of lncRNA-PVT1 hinders the progression of thrombocytopenia. Also, miR-146a has been documented to be implicated in the etiology of autoimmune thyroid diseases [26]. miR-146a is known to exert negative regulation on the inflammatory response, innate immune, and antiviral pathway [27]. A prior study conducted by Lu et al. [13] showed that lncRNA-PVT1 was declined in Tregs during the rejection process, and is inversely interrelated with the expression of miR-146a. Consistent with this evidence, our study found that peripheral blood lncRNA-PVT1 expression was abated and miR-146a expression was hoisted in HT patients, and they were adversely interrelated. Furthermore, peripheral blood lncRNA-PVT1 and miR-146a were independent influencing factors in HT development.

Decreased expression of lncRNA-PVT1 leads to Treg/Th17 dysfunction through the NOTCH pathway in immune thrombocytopenia [12]. miR-146a-5p deletion has been unraveled to attenuate T cell activation and Th17/Treg dysfunction [28]. Innovatively, our study demonstrated that peripheral blood lncRNA-PVT1 was inversely correlated with IL-17, IL-23, and IL-6 levels, and favorably linked with IL-10 level in HT patients. Peripheral blood miR-146a level was positively relevant to IL-17, IL-23, and IL-6 levels, and adversely correlated with the IL-10 level in HT patients. We hypothesized that pathological factors in the internal environment of HT patients led to the downregulation of lncRNA-PVT1. LncRNA-PVT1 increases miR-146a expression by targeting and regulating miR-146a, and miR-146a further affects the expression of its downstream factors [29–31], thereby affecting Th17 and Terg cell numbers and functions, thus demonstrating that lncRNA-PVT1 and miR-146a were significantly correlated with Th17/Treg-related cytokines (IL-17, IL-23, IL-6, IL-10). In summary, peripheral blood lncRNA-PVT1 expression was downregulated and miR-146a expression was upregulated in HT patients, both of which might contribute to the development of HT by affecting the Th17/Treg-related cytokine levels. Subsequently, we plotted ROC curve to probe the diagnostic value of lncRNA-PVT1 and miR-146a in HT, and the results manifested that peripheral blood lncRNA-PVT1 and miR-146a could help HT diagnosis, and the combination of the two had a higher diagnostic value.

Currently, the relationship between lncRNA-PVT1/miR-146a is the subject of numerous studies. A relevant study has demonstrated that lncRNA-PVT1 inhibits miR-146a expression by promoting miR-146a promoter region methylation, which in turn regulates autophagy in Treg cells to inhibit cardiac transplant rejection in mice [13]. LncRNA-PVT1 functions as a miRNA sponge, influencing cell metabolism, cell cycle progression, and development via various pathways [32]. There are various previous studies on the relationship between lncRNA-PVT1/miR-146a and its target genes. LncRNA-PVT1 has been shown to up-regulate cyclooxygenase-2 mRNA expression by binding to miR-146a [33]. Moreover, lncRNA-PVT1 downregulates miR-146a expression by enhancing DNA methyltransferase activity, leading to the methylation of the CpG island in the miR-146a precursor, consequently impacting tumor cell growth [34]. Furthermore, mechanistic investigations on HT have revealed that LINC01061 promotes the development of autoimmune thyroid disease by increasing miR-612-mediated BRD4 expression [35]. However, studies on the relationship between lncRNA-PVT1/miR-146a and its target genes in HT patients are still of high application value. We will continue to focus on this direction in subsequent studies with the aim of gaining a more profound understanding of the molecular mechanisms underlying the low expression of lncRNA-PVT1 and the high expression of miR-146a in HT. Besides, we will further investigate the relationship between lncRNA-PVT1/miR-146a and its target genes, as well as the regulatory mechanisms in vitro.

## Conclusion

To sum up, this study highlighted that lncRNA-PVT1 and miR-146a levels were interrelated with Th17/Treg cytokine imbalance, and that the combination of the two had high diagnostic efficacy for HT, thereby offering valuable insights for the clinical diagnosis of HT. Nevertheless, this article has several important limitations to consider. Further investigation is required to determine if lncRNA-PVT1 and miR-146a also influence Th17/Treg-related transcription factors, indirectly contributing to Th17/Treg imbalance through the modulation of other immune cells, or are influenced by gene polymorphisms of lncRNA-PVT1 and miR-146a. Meanwhile, we will conduct a multicenter study, augment the sample size, and incorporate matched controls, thereby enhancing the sensitivity of the test and the confidence of the obtained results.

## Data Availability

The data that support the findings of this study are available from the corresponding author upon reasonable request.
